# Enhancement of anodic current attributed to oxygen evolution on α-Fe_2_O_3_ electrode by microwave oscillating electric field

**DOI:** 10.1038/srep35554

**Published:** 2016-10-14

**Authors:** Fuminao Kishimoto, Masayuki Matsuhisa, Shinichiro Kawamura, Satoshi Fujii, Shuntaro Tsubaki, Masato M. Maitani, Eiichi Suzuki, Yuji Wada

**Affiliations:** 1 Department of Applied Chemistry, Graduate School of Science and Engineering, Tokyo Institute of Technology. E4-3, 2-12-1, Ookayama, Meguro-ku, Tokyo 152-8552, Japan; 2 Research Fellow of Japan Society for the Promotion of Science, Kojimachi Business Center Building, 5-3-1 Kojimachi, Chiyoda-ku, Tokyo 102-0083, Japan; 3 Department of chemical science and engineering, School of materials and chemical technology, Tokyo Institute of Technology, E4-3, 2-12-1, Ookayama, Meguro-ku, Tokyo 152-8552, Japan; 4 Department of Information and Communication Systems Engineering, Okinawa National College of Technology, 905 Henoko, Nago-shi, Okinawa 905-2192, Japan

## Abstract

Various microwave effects on chemical reactions have been observed, reported and compared to those carried out under conventional heating. These effects are classified into thermal effects, which arise from the temperature rise caused by microwaves, and non-thermal effects, which are attributed to interactions between substances and the oscillating electromagnetic fields of microwaves. However, there have been no direct or intrinsic demonstrations of the non-thermal effects based on physical insights. Here we demonstrate the microwave enhancement of oxidation current of water to generate dioxygen with using an α-Fe_2_O_3_ electrode induced by pulsed microwave irradiation under constantly applied potential. The rectangular waves of current density under pulsed microwave irradiation were observed, in other words the oxidation current of water was increased instantaneously at the moment of the introduction of microwaves, and stayed stably at the plateau under continuous microwave irradiation. The microwave enhancement was observed only for the α-Fe_2_O_3_ electrode with the specific surface electronic structure evaluated by electrochemical impedance spectroscopy. This discovery provides a firm evidence of the microwave special non-thermal effect on the electron transfer reactions caused by interaction of oscillating microwaves and irradiated samples.

Microwaves have potential abilities to enhance chemical reactions caused by microwave thermal effects^1^,^2^,^3^,^4^,^5^,^6^,^7^,^8^, and non-thermal effects^9^,^10^,^11^,^12^,^13^,^14^ called as ‘special effects’. The microwave thermal effects can be realized by applying the characteristics of microwave heating such as, rapid heating^1^ and substance-selective heating^4^, which can be attributed to the mechanism of microwave heating induced by the interaction of oscillating electromagnetic fields with substances. On the other hand, the non-thermal effects are still in veil and need to be investigated to clarify its occurrence and mechanism.

Microwave special effects observed in the organic synthesis field have been extensively studied. Some evidences of the microwave special effects on enantioselective organic reactions in a homogeneous system have been reported^10^,^11^. Even though microwave special effects on reactions on solid surfaces were also observed in several systems^12^,^13^, definitive demonstrations have never been reported because the reaction mechanism is more complex than the organic reactions in homogeneous phases. Recently, our group reported that the photoinduced electron transfer reaction from CdS quantum dots to organic molecules was accelerated by microwave special effects^14^. We estimated the electron transfer rates by a fluorescence quenching of CdS quantum dots due to the electron transfer reaction. However, since this was one and only observation of the microwave special effects in electron transfer reactions and just led to the speculated mechanism, more simple and general observations of the microwave non-thermal effects on the reactions at solid surface have been desired. While there are reports on the microwave special effects on specific reactions on solid surface^15^,^16^, a generalization of the special effects is a high-priority issue in the field of microwave chemistry.

In this report, we focused on an electrochemical method to generalize the microwave enhancement of electron transfer reaction on the solid surface, since we can appreciate an advantage, i.e., to enable quantitatively detecting the electron transfer under manipulated potential. The electrochemical method under microwave irradiation was investigated by Marken’s group. They observed the electrochemical redox reactions of ferrocyanide ions or ferrocene molecules using a platinum disc electrode, and demonstrated that the maximum reduction current was increased under 0.6 second pulsed microwave irradiations^17^,^18^. Unfortunately they failed in observing the microwave special effects, since these electrochemical redox reactions are diffusion-limited. The increased reduction current was not attributed to the microwave special effects in the electron transfer reaction, but caused by the microwave thermal effects on the substance diffusion and convection flow around the electrode.

We have observed the electrochemical oxidation of water to produce dioxygen on an α-Fe_2_O_3_-deposited fluorine-doped tin oxide (α-Fe_2_O_3_/FTO) electrode under 2.45 GHz microwave irradiation in a repeated pulse mode of 1 sec. A rate-determining step of the oxidation reaction of water is the electron transfer from α-Fe_2_O_3_ to water because four electrons should be subtracted from water to produce oxygen^19^,^20^,^21^,^22^,^23^. Therefore, a change of the current density of water oxidation can be directly related to the alternation of the rate of the electron transfer. In order to investigate the microwave special effects, the temperature alternation at the reaction field by microwaves should be carefully discussed. The temperature was measured by a fiber-optic thermometer which was placed close to the α-Fe_2_O_3_/FTO electrode. Furthermore, the surface temperature of the α-Fe_2_O_3_/FTO electrode under microwaves was also measured by an infrared radiation thermometer. These accurate temperature measurements confirmed that the attribution of the enhancement of the current density of water oxidation to the microwave special effect on the electron transfer reactions.

## Results and Discussion

α-Fe_2_O_3_/FTO working electrodes prepared by an electrodeposition method^24^ were characterized by X-ray diffraction method (Fig. S1a). After a sintering process, the diffraction peaks attributed to α-Fe_2_O_3_ were observed. The surface structure of sintered α-Fe_2_O_3_ was observed by a scanning electron microscope (Fig. S1b). α-Fe_2_O_3_ was observed as an aggregation of nanoparticles with a size of 10~100 nm. The thickness of the α-Fe_2_O_3_ layer was determined as ca. 700 nm by DektakXT Stylus Profilometer.

**Figure 1:**
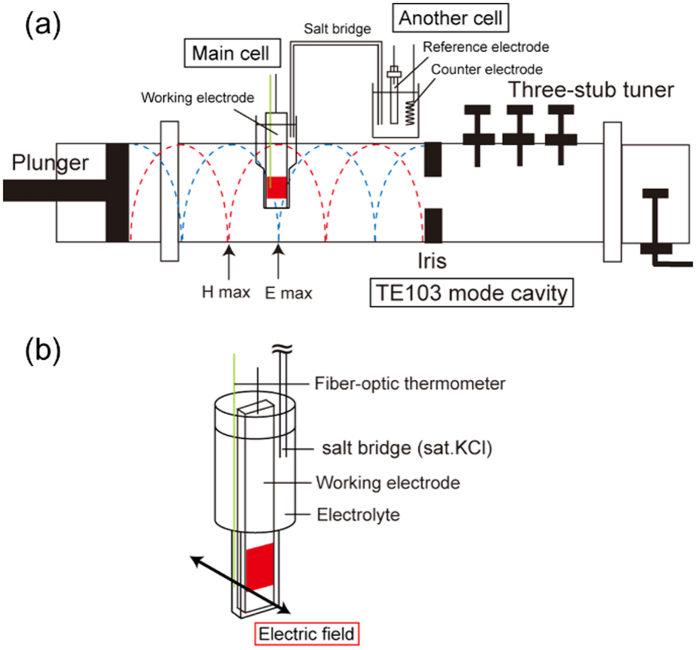
Experimental setup of the electrochemical measurements. (**a**) The microwave irradiation cavity and the positions of the electrochemical cells. The main cell was introduced into the microwave cavity at E max (electric field) or H max (magnetic field). (**b**) Detailed illustration of the main cell. A fiber-optic thermometer was introduced close to the electrode.

The setup of an electrochemical measurement was setup in 2.45 GHz waveguide-type single mode microwave cavity as shown in Fig. 1. A main cell was placed into the microwave resonator while another cell connected electrochemically with the main one through a salt bridge was put outside of the microwave resonator. Microwaves were irradiated only to the main cell containing a working electrode as shown in Fig. 1a. Figure 1b shows a detail setup of main cell. The main cell was made of quartz which was transparence to microwaves. The tip section of the main cell targeted by microwave irradiation was 5 mm-thick and 50 mm-long. A fiber-optic thermometer was introduced close to the electrode. More detailed explanations are described in the method section.

**Figure 2:**
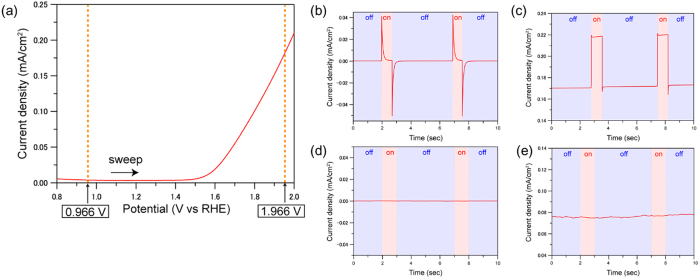
The microwave effects on oxidation current of water. (**a**) Linear sweep voltammogram of the α-Fe_2_O_3_/FTO electrode immersed in 0.1 M NaOHaq. The scan rate was 100 mV/s. Potentiostatic ammperometry at 0.966 V vs. RHE (**b**) and 1.966 V vs RHE (**c**) under pulsed microwave irradiation. The electrochemical cell was located at the maximum point of oscillating electric field. Potentiostatic ammperometry at 0.966 V vs. RHE (**d**) and 1.966 V RHE (**e**) with pulsed microwave irradiation. The electrochemical cell was located at the maximum point of oscillating magnetic field.

The onset potential of the anodic current observed for the α-Fe_2_O_3_/FTO electrode immersed in 0.1 M NaOHaq was ca. 1.5 V vs. RHE (Fig. 2a). The onset can be attributed to the occurrence of the oxidation reaction of water to dioxygen^25^. Figure S2 shows the potentiostatic ammperometry of the α-Fe_2_O_3_/FTO electrode immersed in 0.1 M NaOHaq. The potential of the electrode was set at the equilibrium (rest) potential in the first 10 second. When the potential of the electrode was changed to 1.966 V vs. RHE, the anodic current density was instantaneously increased, and subsequently stabilized at ca. 0.26 mA cm^−2^, showing that the oxidation reaction of water to oxygen should occur continuously. Hereinafter, the main cell was irradiated with microwave irradiation in the pulse mode of 1 second in order to investigate the microwave effects on the oxidation of water.

Figure 2b,c show the result of the potentiostatic ammperometry of the electrode positioned at the antinode of the oscillating electric field where the electric field is maximum in the cavity (E max) under 1 second-pulsed irradiation. The vibrating electric field was oriented in the vertical direction against the α-Fe_2_O_3_/FTO electrode. When the applied potential of the α-Fe_2_O_3_/FTO electrode was set at 0.966 V vs. RHE at which no anodic current was observed, the spike-like temporal current rises and falls were observed at the moment of the introduction and removal of microwaves, respectively. After the temporal current rises and falls, the current density was steeply recovered to 0.00 mA cm^−2^. Moreover, the integrated area of the positive spikes measured at the introduction of microwaves coincided with that of the negative spikes at the end of microwave irradiation. Therefore, these current changes should not be attributed to the external chemical reactions, but to the change of the carrier balances at the interfaces, such as between α-Fe_2_O_3_ and FTO, or α-Fe_2_O_3_ and the electrolyte. The spike-like temporal current rises were not observed in the FTO electrode without α-Fe_2_O_3_ (Fig. S3(c)). This information strongly supports the attribution of the spike-like temporal current rises to the change of the carrier balances around the α-Fe_2_O_3_.

When the potential of the electrode was set at 1.966 V vs. RHE at which the anodic current due to oxidation of water to dioxygen was substantially observed, the rectangular waves were observed under pulsed microwave irradiation (Fig. 2c). The current density was raised from 0.17 mA cm^−1^ to ~0.22 mA cm^−1^ simultaneously with microwave irradiation. Moreover, the current density was kept at ~0.22 mA cm^−1^ during microwave irradiation. When the microwave irradiation was switched off, the current density went back to 0.17 mA cm^−1^. We tested the electrode properties at the potential of 1.666 V vs. NHE (Fig. S4). The rectangular waves were also observed under pulse microwave irradiation, and were smaller than those at the potential of 1.966 V vs. NHE. Therefore, the steady increment of the anodic current density should be attributed to the enhancement of oxygen evolution reaction on the surface of the α-Fe_2_O_3_/FTO electrode by the oscillating electric field of microwaves.

On the other hands, Fig. 2d,e show the result of potentiostatic ammperometry of the electrode positioned at the antinode of the oscillating magnetic field where the magnetic field was maximum in the cavity (H max). The electrode was oriented in the vertical direction against the vibrating magnetic field. No changes in the current density by microwave irradiation were observed at either potentials, 0.966 V vs. RHE and 1.966 V vs. RHE. We have obtained an important conclusion that the microwave enhancement on the oxidation current of water was induced only by microwave oscillating electric field, but not by oscillating magnetic field.

Figure S3 shows the results of the electrochemical measurements of an FTO electrode without α-Fe_2_O_3_. The onset potential of the FTO electrode immersed in 0.1 M NaOH aq was observed at ca. 1.7 V vs. RHE (Fig. S3a). Under pulsed microwave oscillating electric field, the anodic current density attributed to water oxidation to dioxygen showed no change. (Fig. S3b) Therefore, we can attribute the rectangular waves observed in the electrochemical measurement of α-Fe_2_O_3_/FTO electrode under pulsed microwave irradiation to the interaction of microwave oscillating electric field with α-Fe_2_O_3_.

**Figure 3:**
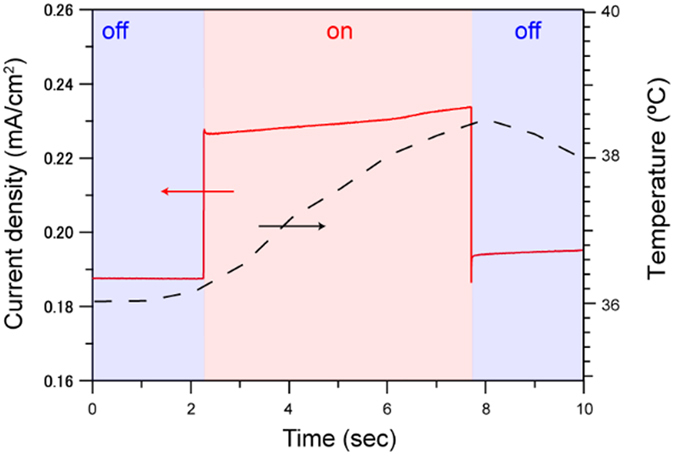
Potentiostatic ammperometry at 1.966 V vs. RHE under microwave irradiation for 6 seconds. The main cell was located at the maximum point of oscillating electric field. The dotted black line shows the temperature profile of measured by a fiber-optic thermometer positioned close to the electrode.

When the main cell was irradiated by microwaves in the pulse mode with 6 seconds, the enhanced current density under microwaves was gradually increased after the sudden increase observed at the initiation of the pulse. In Fig. 3, the irradiation of microwave oscillating electric field was initiated at 2 second, and then the current density was increased from ~0.188 mA cm^−2^ to ~0.225 mA cm^−2^ instantaneously. After that, the current density was gradually increased to 0.235 mA cm^−2^ during the stable microwave irradiation. Subsequently, the irradiation of microwaves was stopped at 8 second, the current density was decreased to ~0.195 mA cm^−2^ suddenly. The gradual increased current density under microwave irradiation from ~0.188 mA cm^−2^ to ~0.195 mA cm^−2^ can be attributed to the temperature elevation of the electrode and the electrolytes by microwaves. The temperature profile of electrolytes under microwaves measured by the fiber-optic thermometer is shown in Fig. 3. The liquid phase was heated from 36.2 °C to 38.4 °C under microwave irradiaiton for 6 second.

**Figure 4:**
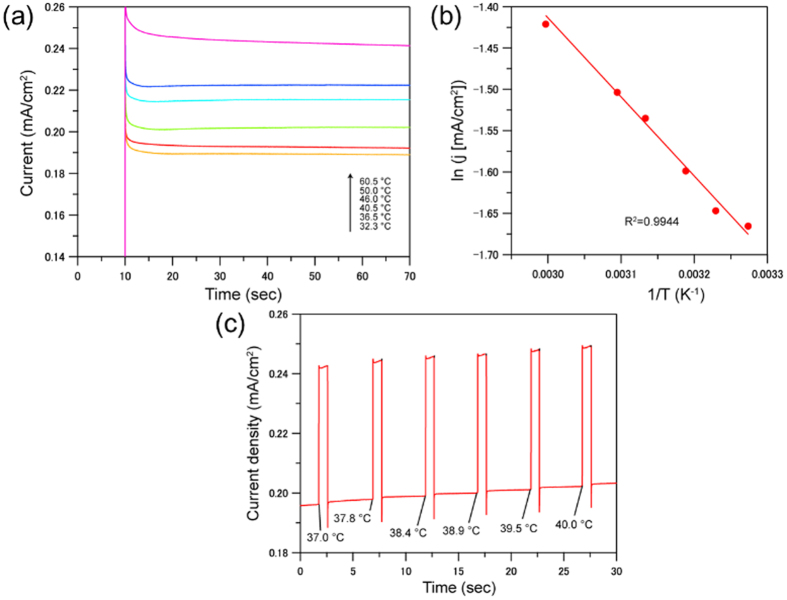
Temperature dependence of the anodic current. (**a**) Temperature dependence of the anodic current. The electrode was set at the rest potential in the initial 10 seconds, subsequently the electrode was set at 1.966 V vs. RHE. (**b**) Plots of the logarithm of the current density at 70 second against the reciprocal temperatures. The plots are fitted as the displayed solid line. (**c**) Change of the anodic current under repeated microwave irradiation. The temperatures of the liquid phase are shown below the background current.

The anodic current density should be affected by the temperature of the electrode. Therefore, it is important to carefully discuss the measured enhancement in the anodic current under microwave irradiation, in other words, whether the enhancement was caused by the temperature alternation or by the microwave special effects. Figure 4a shows the temperature dependence of the anodic current density observed at the applied potential of 1.966 V vs. RHE. This experiment was carried out using the main cell heated under conventional heating using a heating blocks. The temperature of the liquid phase was measured by a fiber-optic thermometer. When the potential was applied at 10 second, the steady state current density was dependent on the temperature of the liquid phase. In this case, the current change was fully attributed to the effect of temperature alternation. The logarithm of the anodic current density observed at 70 second was linearly related to the inverse of the temperature (Fig. 4b). Let us ensure that the enhancement in the anodic current observed under microwave irradiation is not caused by the temperature rise of the surface of the electrode. Figure 4c shows the enhancement in the anodic current under repeated microwave irradiation. The temperatures of the liquid phase shown below the background current indicate the rise of the liquid phase during the experiment due to the heat formation by microwaves. The slow rise in the background current should be due to this temperature rise of the liquid phase (approximately 3 °C with 6 time pulsed microwave irradiation in 30 sec). If the increment in the anodic current observed under microwave irradiation (ca. 0.05 mA/cm^2^) was attributed to the temperature rise, the value of the temperature rise should be estimated as 60 °C, meaning that the surface temperature of α-Fe_2_O_3_ was raised and lowered by 20 °C within at least 0.02 second. This cannot be reality in the present experiment. Then, we concluded that the increment of anodic current under microwave irradiation should be induced by the microwave special effect.

The surface temperature of the α-Fe_2_O_3_/FTO electrode was measured by an infrared radiation thermometer. In this measurement, the electrode was directly introduced into the microwave cavity without a reaction vessel and an electrolyte (Fig. S5a). The surface temperature was raised from 25 °C to 31 °C under microwave irradiation for 60 second (Fig. S5b). Therefore we can exclude a possibility that the surface temperature of the electrode is raised up much, causing the enhancement of the anodic current under microwave irradiation.

Here, we need to note the reproducibility of these microwave enhancement in the oxidation current of water. We prepared seven electrodes of α-Fe_2_O_3_/FTO by the same electrodeposition method, but could not observe the enhancement for all the samples. The current density of each electrode at 1.966 V vs. RHE under 1 second pulsed microwave irradiation is shown in Fig. S6. Hereinafter, these electrodes are identified as sample 1~7. Sample 1 was used in the temperature alternation experiment, giving the data shown in Fig. 4 and Fig. S5. Sample 1, 2 and 3 showed the rectangular waves under pulsed microwaves, while sample 4~7 gave no response under microwave irradiation. We attempted to find the difference in the electrochemical parameters among the samples by an electrochemical impedance spectroscopy.

**Figure 5:**
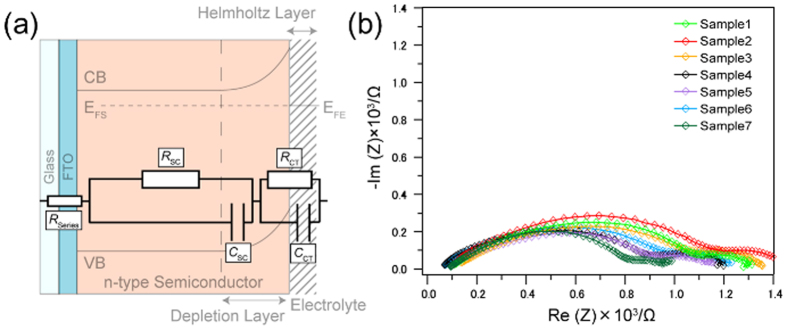
Analysis of the electronic structure of α-Fe_2_O_3_/FTO electrode by electrochemical impedance spectroscopy. (**a**) Supposed equivalent circuit of α-Fe_2_O_3_/FTO electrode. (**b**) The Nyquist plots of the α-Fe_2_O_3_/FTO electrode.

The equivalent circuit shown in Fig. 5a was used for modeling the interface between the electrolyte and the α-Fe_2_O_3_/FTO electrode^26^. This equivalent circuit consists of two capacitors, *C*_SC_ referred from the depletion layer at the surface of α-Fe_2_O_3_ and *C*_H_ attributed to an electrical double layer (Helmholtz layer), and three resistance, *R*_series_ caused from the resistance of FTO, electrolyte, and electrical cables, *R*_SC_ attributed to the resistance of bulk α-Fe_2_O_3_, *R*_CT_ attributed to the resistance at the interface of α-Fe_2_O_3_ and electrolyte. Since the electronic process in the bulk semiconductor is faster than that in the electrolyte, the electrical response in the lower frequency region can be attributed to the components at the electrical double layer and the interface of α-Fe_2_O_3_ and electrolyte, while the response in the higher frequency region can be attributed to the components in the semiconductor^27^,^28^.

Figure 5b shows the Nyquist plots of each electrode under applied potential of 1.8 V vs. NHE, giving the each ohmic value and capacitance by the mathematical fitting with the equivalent circuit shown in Fig. 5a as summarized in Table 1. The *C*_SC_ values of sample 1~3 exhibiting the enhancement in the anodic current under microwave irradiation were relatively low (5.94~19.23 μF) compared to those of sample 4~7 (23.41~48.49 μF), showing no enhancement. The *C*_SC_ value reflects the surface electronic structure of α-Fe_2_O_3_, furthermore the difference of the *C*_SC_ values of α-Fe_2_O_3_ reflects the amount of surface defect site^29^. Previously, it was reported that the microwave properties of the metal oxides had a dependence on the amount of oxygen vacancies^30^. Therefore, we speculate that the enhancement in the anodic current under microwave irradiation is related with the surface electronic structure of α-Fe_2_O_3_, particularly the distribution of the surface defect state.

In summary, we have discovered that the anodic current of α-Fe_2_O_3_ electrochemically deposited on FTO substrate was steadily enhanced by 2.45 GHz microwave irradiation. The enhancement was strongly related to the interaction between the surface electronic structures of α-Fe_2_O_3_ and microwave oscillating electric field. We have concluded that the enhancement in the anodic current on α-Fe_2_O_3_ should be attributed to the enhanced oxidation of water into dioxygen under microwave irradiation. This is a clear experimental evidence of microwave special effects on the electron transfer reactions. This demonstration of the microwave special effects in extremely simple electrochemical systems leads us to the in-depth investigation of physical insights of these special effects.

## Methods

### Synthesis of α-Fe_2_O_3_ electrodes

An electrodeposition of α-Fe_2_O_3_ on FTO was carried out using an aqueous solution containing 0.02 M FeCl_2_ · 4H_2_O (Wako Pure Chemical Industries, Ltd.). Deionized water (resistivity 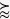 18.2 MΩ · cm) purified with an ultrapure water production system (Direct-Q UV 3, Merck Millipore Corporation) was used to prepare all solutions used in this study. In the electrodeposition method, a standard three-electrode setup in an undivided cell was used. FTO substrates (resistance ~8–12 Ω) were used as the working electrode, the coiled platinum wire as a counter-electrode and an Ag/AgCl (in sat. KCl solution) electrode as a reference electrode. FTO substrates were cleaned by ultrasonic rinsing in an aqueous solution containing Contaminon N (Wako Pure Chemical Industries, Ltd.), ultrapure water, and ethanol, for 30 min each time. After drying, those were cleaned by a UV/O_3_ cleaning process for 15 min. The electrodeposition method was potentiostatically performed at 75 °C for 12 min by applying 1.2 V vs Ag/AgCl reference electrode under gentle stirring (an average deposition current density was ~0.40 mA cm^−2^). After each deposition, the resulting film was thoroughly rinsed with deionized water, and dried with a gentle stream of nitrogen gas. And then as-deposited films were annealed in atmosphere at 520 °C for 2 h after heating at a rate of 2 °C/min.

### Material characterization

X-ray diffraction (XRD) spectra were obtained using Benchtop X-ray diffractometer Miniflex (Rigaku Corporation and its Global Subsidiaries) with bent monochromated Cu Ka radiation. Scanning electron microscopy (SEM) images were collected by Hitachi S-5500 scanning electron microscope (Hitachi High-Technologies Corporation) operated at 3 kV. Thickness of films were measured using DektakXT Stylus Profilometer (Bruker Corporation).

### Details of the TE 103 mode 2.45 GHz microwave instrumentation setup

The 2.45 GHz microwave irradiation was performed using a waveguide type microwave resonator (Fuji Electronic Industrial Co. Ltd). Moving the plunger at the end of the waveguide, the standing waves of TE103 mode were made by controlling the interference of the incident and reflected waves. The electric field component of this standing waves was made out of phase with the magnetic one by 90 degrees. Hence, this apparatus could separate the electric field (E field) and the magnetic field (H field) distribution in the waveguide. The main cell for the electrochemical measurements was placed into the microwave resonator and another cell was placed outside of the microwave resonator to avoid its exposure to microwaves. Only the main cell was irradiated by microwaves, containing the working electrode as shown in Fig. 1a. Figure 1b shows the detailed setup of the main cell. The main cell made of quartz was transparent to microwaves. The tip section of main cell was 5 mm thick and 50 mm long in its shape. A fiber-optic thermometer and a salt bridge were placed close to the α-Fe_2_O_3_/FTO electrode and contacted with aqueous solutions of 0.1 M NaOH electrolyte (pH = 13). The salt bridge was used to link the main cell and another cell. The coiled platinum wire as a counter-electrode and an Ag/AgCl (in sat. KCl solution) electrode as a reference electrode were placed into the another cell to prevent the temperature and reference potential of those electrodes from changing. In this experiment, the IR drop resulting from the resistance of the solution between the reference electrode and the working electrode was not taken into account. As the measuring conditions under microwave irradiation were checked without microwave irradiation, the IR drop should not affect the results and discussion in this work.

### Procedure for microwave-activated electrochemical measurements

*I-V* property was measured by biasing electrodes using a potentiogalvanostat (SP-150, Bio-Logic Science Instruments). The potentiostat was controlled by EC-lab (Bio-Logic Science Instruments). The reference electrode was regularly calibrated vs an Ag/AgCl electrode, and all potentials were converted to the reversible hydrogen electrode (RHE) scale by the equation 1;


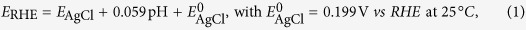


where *E*_RHE_ is the potential of the reversible hydrogen electrode, *E*_AgCl_ is the measured potential relative to the saturated Ag/AgCl electrode, and *E*^0^_AgCl_ is the potential of the saturated Ag/AgCl electrode. In this experiment, the current profiles of α-Fe_2_O_3_ electrodes were investigated by the potentiostatic ammperometry method at the applied potential necessary for the oxygen evolution reaction as shown equation 2.


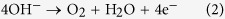


The pulsed microwave irradiation was performed while the anodic current was observed attributed to this oxygen evolution reaction (2). The applied microwave power was unified at 30 W in all the experiment. As shown Fig. 1a, the main cell was placed either at the position of maximum E field density or at the maximum H field within the waveguide. As shown in Fig. 1b, the oscillating direction of the E field or H field was defined so as to apply perpendicularly relative to the electrodes.

## Additional Information

**How to cite this article**: Kishimoto, F. *et al*. Enhancement of anodic current attributed to oxygen evolution on α-Fe_2_O_3_ electrode by microwave oscillating electric field. *Sci. Rep.* **6**, 35554; doi: 10.1038/srep35554 (2016).

## Supplementary Material

Supplementary Information

## Figures and Tables

**Table 1:** The calculated values of the resistances and the capacities from Nyquist plots in Fig. 4b by analyzed on the basis of the equivalent circuit of Fig. 4(a).

Sample	*R*_SC_ (×10^2^ Ω)	C_SC_ (μF)	*R*_CT_ (×10^2^ Ω)	C_SC_ (mF)	Microwave response*
1	9.83	9.54	2.52	2.30	○
2	10.67	19.23	1.43	5.45	○
3	8.51	5.94	2.41	2.72	○
4	10.13	23.41	1.44	11.88	×
5	10.12	23.73	1.87	10.61	×
6	9.28	4142	6.97	19.24	×
7	7.48	48.49	4.30	28.53	×

^*^Circle means that the rectangular waves in anodic current under microwaves were observed.
