# Supplementary material for: Enhancement of anodic current attributed to oxygen evolution on α-Fe2O3 electrode by microwave oscillating electric field
Source: Sci Rep. 2016 Oct 14;6:35554. doi: 10.1038/srep35554 (PMC5064412; doi:10.1038/srep35554)
Supplement: Supplementary Information [file srep35554-s1.pdf]

Supplementary Information

Enhancement of anodic current attributed to oxygen evolution on α-Fe_2O_3 electrode by microwave oscillating electric field.

Fuminao Kishimoto[1, 2]*, Masayuki Matsuhisa[3], Shinichiro Kawamura[1], Satoshi Fujii[3, 4], Shuntaro Tsubaki[3], Masato M. Maitani[3†], Eiichi Suzuki[3], Yuji Wada[3]*.

1. Department of Applied Chemistry, Graduate School of Science and Engineering, Tokyo Institute of Technology.
E4-3, 2-12-1, Ookayama, Meguro-ku, Tokyo 152-8552 (Japan)
E-mail: yuji-w@apc.titech.ac.jp; kishimoto.f.aa@m.titech.ac.jp
2. Research Fellow of Japan Society for the Promotion of Science.
3. Department of chemical science and engineering, School of materials and chemical technology, Tokyo Institute of Technology.
4. Department of Information and Communication Systems Engineering, Okinawa National College of Technology, 905 Henoko, Nago-shi, Okinawa 905-2192, Japan
†Present address; Research Center for Advanced Science and Technology, The University of Tokyo, 4-6-1, Komaba, Meguro-ku, Tokyo, 153-8904 Japan.

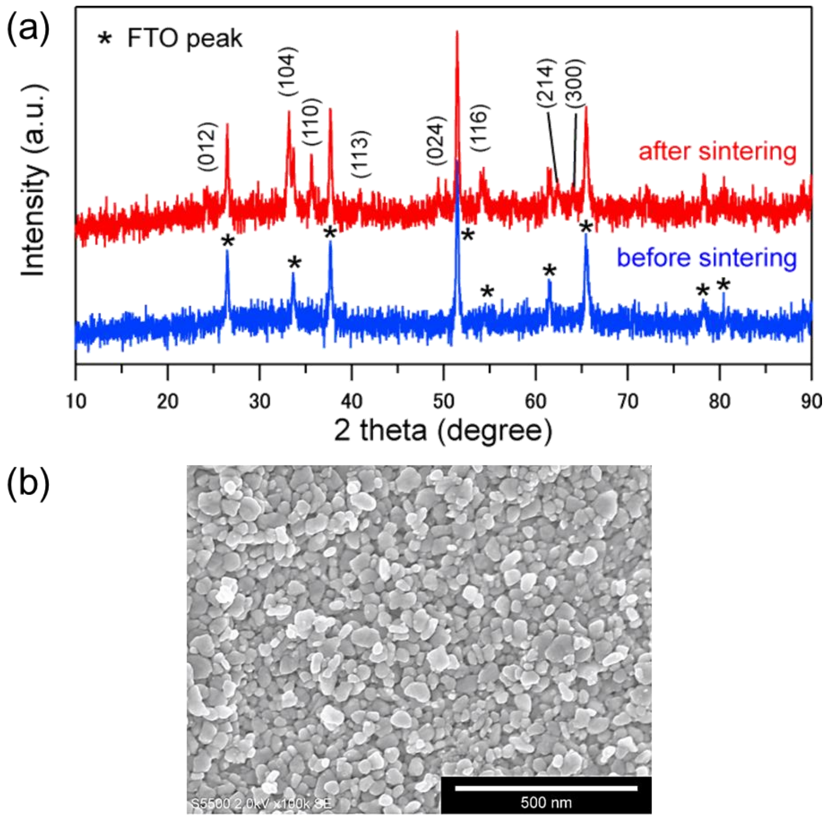

Figure S1. (a) XRD patterns of α-Fe_2O_3 electrode fabricated on FTO. (b) SEM image of the electrode after sintering at 520 °C for 2 h.

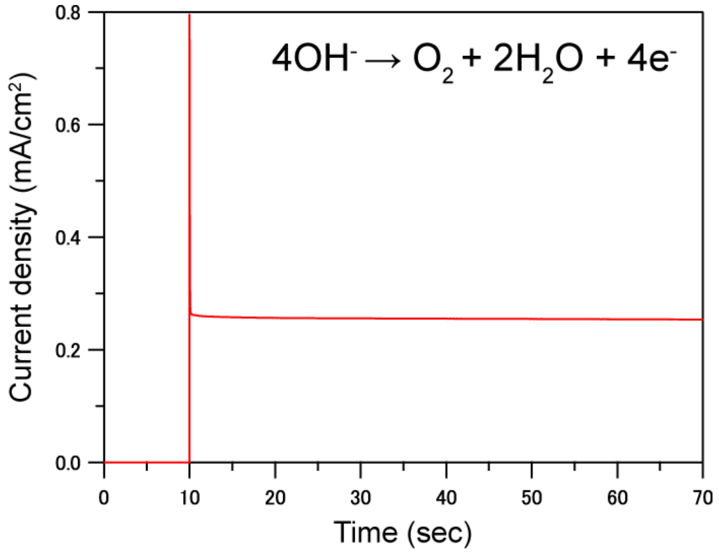

Figure S2. Potentiostatic ammperometry of α-Fe_2O_3 electrode. At 10 second, the electrode was set at 1.966 V vs. RHE. The oxidation current was stable in this time range.

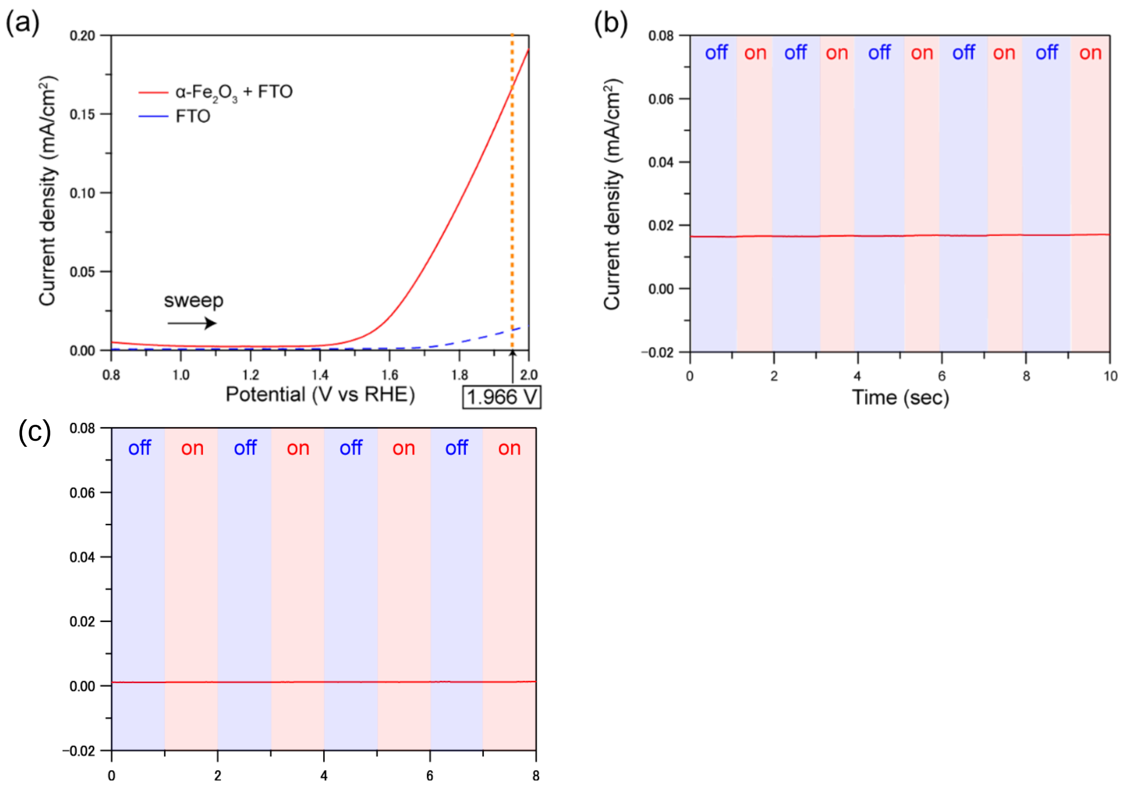

Figure S3. (a) Linear sweep voltammogram (LSV) of FTO electrode immersed in 0.1 M NaOHaq. The scan rate was 100 mV/s. (b) Potentiostatic ammperometry at 1.966 V vs. RHE with pulsed microwave irradiation. The electrochemical cell was located at the maximum point of alternating electric field. (c) Potentiostatic ammperometry at 0.966 V vs. RHE with pulsed microwave irradiation. The electrochemical cell was located at the maximum point of alternating electric field.

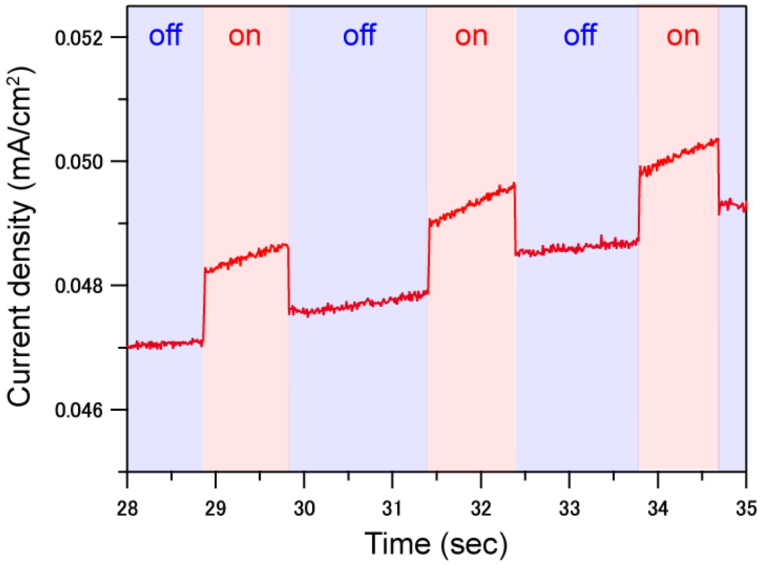

Figure S4. The microwave effects on oxidation current of water by using the α-Fe_2O_3/FTO electrode. Potentiostatic ammperometry at 1.666 V vs. RHE with pulsed microwave irradiation. The electrochemical cell was located at the maximum point of oscillating electric field.

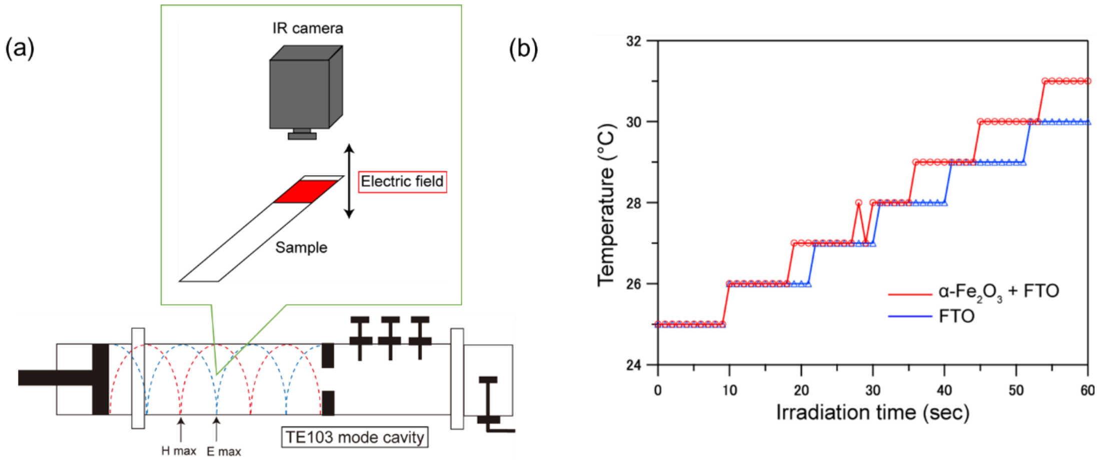

Figure S5. Direct temperature measurement of the electrode under microwave irradiation by means of IR radiation thermometer. (a) Illustration of the temperature measurement setup. The electrode was introduced into the microwave cavity, and the surface temperature was directly measured by external IR camera. (b) The temperature variation of the electrode under microwave irradiation.

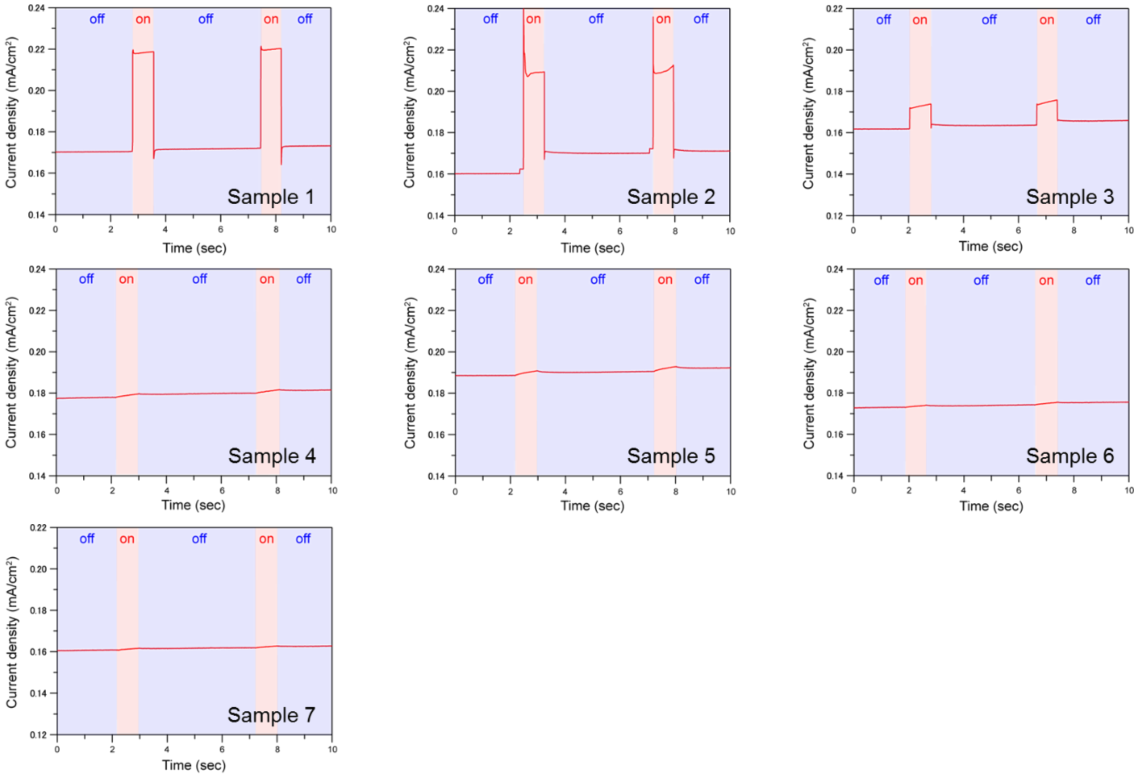

Figure S6. The response of the anodic current induced by microwave irradiation. Even though we prepared seven electrodes by the same electrodeposition method, the response of each electrode was different each other.
